# In Vitro and In Vivo Crosstalk between Type I IFN and IL-8 Responses in SARS-CoV-2 Infection

**DOI:** 10.3390/microorganisms11112787

**Published:** 2023-11-16

**Authors:** Mirella Biava, Stefania Notari, Germana Grassi, Licia Bordi, Eleonora Tartaglia, Chiara Agrati, Eleonora Cimini, Giuseppe Sberna, Emanuele Nicastri, Andrea Antinori, Enrico Girardi, Francesco Vaia, Fabrizio Maggi, Eleonora Lalle

**Affiliations:** 1 Laboratory of Virology and Biosafety Laboratories, National Institute of Infectious Diseases “L. Spallanzani”, IRCCS, 00149 Rome, Italygiuseppe.sberna@inmi.it (G.S.);; 2 Cellular Immunology Laboratory, National Institute of Infectious Diseases “L. Spallanzani”, IRCCS, 00149 Rome, Italy; 3 Unit of Pathogen Specific Immunity, Ospedale Bambino Gesù, IRCCS, 00146 Rome, Italy; 4 Clinical and Research Department, National Institute of Infectious Diseases “L. Spallanzani”, IRCCS, 00149 Rome, Italy; 5 Scientific Direction, National Institute of Infectious Diseases “L. Spallanzani”, IRCCS, 00149 Rome, Italy; 6 General and Health Management Direction, National Institute of Infectious Diseases “L. Spallanzani”, IRCCS, 00149 Rome, Italy

**Keywords:** IFN-I, IL-8, SARS-CoV-2, crosstalking, antagonism, COVID-19

## Abstract

COVID-19 patients show characteristic over-expression of different cytokines that may interfere with the interferon (IFN) response, delaying its production. Within the overexpressed cytokines, IL-8 plays a key role, and it may impede IFN-I activation. PBMC from eight healthy donors were exposed to 2019-nCoV/Italy-INMI1 isolate and supernatants/cells were collected at different time points; the production of either IFN-alpha or IL-8 was assessed. The same analysis was performed on plasma samples obtained from 87 COVID-19 patients. Antagonism between IFN-alpha and IL-8 was observed, since in those PBMC with medium or high IL-8 levels, IFN-α levels were low. The same scenario was observed in SARS-CoV-2-infected patients that were divided into three groups based on IL-8 low, medium and high levels; the correlation between low levels of IFN-α and high levels of IL-8 was statistically significant in both the IL-8 medium and IL-8 high group. Overall, our results showed a crosstalk/antagonism between IL-8 and IFN-alpha in PBMC from healthy donors challenged with SARS-CoV-2 and inversely proportional IFN-alpha levels to IL-8 concentrations detected in plasma samples from COVID-19 patients, suggesting that the impairment of the innate immune response in COVID-19 patients may be linked to a dysregulated cytokine response, namely through IL-8 production.

## 1. Introduction

The Coronaviridae family is formed by positive, single-stranded RNA viruses and includes Coronaviruses (CoVs). CoVs target different species, including mammals and humans. Recently, a new coronavirus was identified in China and was named Severe Acute Respiratory Syndrome Copronavirus-2 (SARS-CoV-2). This virus was responsible for the 2019 Coronavirus Disease (COVID-19) and in March 2020, the WHO made the assessment that SARS-CoV-2 could be characterized as a pandemic. As of March 2023, COVID-19 had caused over 6.8 million deaths from among 760 million confirmed cases [1]. The disease may present in several ways; patients may be asymptomatic or experience mild to severe symptoms. The most frequent manifestations include cough, fever, and headache. On the other hand, severe cases can present a generalized inflammation associated with extended tissue damage and may develop acute respiratory distress syndrome (ARDS), thromboembolism, neurological manifestations, cardiac injury, cytokine storm, and organ failure, which overall can lead to death of the patient.

The fate of COVID-19 is linked to different aspects, some related to the virus (such as the specific variant), and other related to the patient (such as age, ethnicity, vaccination, comorbidities, and genetics) [2,3,4,5].

The innate immune response represents the first line against any invading pathogen, including SARS-CoV-2. Recognition of SARS-CoV-2 invasion starts with the identification of viral pathogen-associated molecular patterns (PAMPs) via diverse pattern recognition receptors (PRRs), leading to their activation and cascading into the antiviral immune response, during which type I and III interferon (IFN) play a key role [6]. The role of IFN response during SARS-CoV-2 infection is not yet fully understood and its role in other coronavirus infections is debated. A dysregulated immune response may play a primary role in severe SARS-CoV-2 infection, which is characterized by delayed IFN type I and decreased IFN type II responses, and high serum levels of proinflammatory cytokines, [7,8,9,10]. On the contrary, a productive induction of IFN-I and -II was evidenced in SARS-CoV infection in peripheral blood mononuclear cells (PBMC). This IFN expression seemed to be untied to viral replication, as it was obtained also by the co-cultivation of normal PBMC with fixed SARS-CoV-infected cells [11]. Regarding the IFN response, there is a paradox in the COVID-19 context. IFNs levels were found to be both increased and decreased in patients with COVID-19, with a consistent aberrant response in severe cases [12,13,14].

On the other hand, a dysregulated immune response may be at the base of the limited percentage of patients that develop severe conditions, such as Cytokines Release Syndrome (CRS) or Macrophage Activation Syndrome (MAS) [15]. In these cases, several cytokines such as IL-1β, IL-6, IL-7, IL-8, IL-9, TNFα, IL-10, IL-17, MCP-1 (monocyte chemoattractant protein-1), GM-CSF and IFN-γ were found to be overexpressed [16,17]. Moreover, the delay in the IFN response results in an uncontrolled viral replication with increased viral load. This high viremia seems to be linked to an ineffective adaptive immune response, which ultimately is unable to fight the infection. Thus, the waste of time is associated with a storm of cytokines, such as IL-1β. -6, -8, -10, -12, -17, TNFα, IFN-γ, MCP-1, IP-10, C-reactive proteins (CRP), CXCL10, D-dimer and ferritin [8,18,19,20]. This immune dysregulation may result in the so-called COVID-19-related cytokine storm (COVID-CSS), which is associated with a negative outcome [18]. 

In COVID-19 patients, within the overexpressed cytokines, IL-8 plays a key role. In fact, IL-8 was recently identified as a sensitive and easily detectable biomarker which can be associated with mild or severe COVID-19 patients [21]. IL-8 is a potent neutrophil chemotactic factor, with a key role in several pathological and physiological conditions. Interestingly, Type I IFNs downregulates IL-8 production in vivo and in vitro in hematopoietic cells, prompting an anti-inflammatory role of IFNs, which may be explained by the reduction of neutrophils through inhibition of their main chemotactic signal [22]. On the contrary, IL-8 has been shown to inhibit the antiviral action of IFN-alpha and to be overexpressed in viral infections [23,24].

In this study, we evaluated the effects of 2019-nCoV/Italy-INMI1 isolate infection on viral replication in human PBMC; induction of innate immune response, namely on the IFN type I and II responses; and modulation of IL-8 expression in both human PBMC and plasma samples from COVID-19 patients. We specifically evaluated the possible cross-talk and/or antagonist effect between IL-8 expression and INF-α response.

## 2. Materials and Methods

### 2.1. Plasma Samples

Eighty-nine unvaccinated COVID-19 patients hospitalized at the National Institute for Infectious Diseases “L. Spallanzani” were included, with a median age of 65 years (range: 14–98 years); thirty-three of them (37.1%) were females.

Plasma samples were collected for diagnostic purposes from March to April 2020. Residual plasma samples were used for research purposes and data from biological samples were used after complete anonymization only.

### 2.2. Virus Stock Preparation

Vero E6 cells (ATCC^®^ Number CRL-1586™) were maintained in Modified Eagle Medium (MEM) supplemented with 10% heat-inactivated Fetal Calf Serum (FCS) at 37 °C in a humidified atmosphere of 5% CO_2_. For virus stock preparation, Vero E6 cells were infected with SARS-CoV-2 2019-nCoV/Italy-INMI1 isolate (GenBank Accession # MT008022) obtained in January 2020 from clinical samples taken from a Chinese tourist. Cell lysates were clarified, aliquoted, and stored at −80 °C until use. Virus titration was performed on Vero E6 cell line by limiting dilution assay; the titer was calculated using the method of Reed & Muench and expressed as tissue culture infectious dose TCID_50_/mL [25]. The virus stock titer was: 10^7.25^ TCID_50_/mL. These experiments were performed in a Biosafety level 3 (BSL3) facility.

### 2.3. In Vitro Infection of Vero E6 Cells 

Vero E6 cells (ATCC^®^ Number CRL-1586™) were maintained in MEM supplemented with 10% heat-inactivated FCS at 37 °C in a humidified atmosphere of 5% CO_2_. Vero E6 cells were exposed to SARS-CoV-2 for 1 h at 37 °C at a multiplicity of infection (MOI) of 0.1. At the end of the adsorption period, cells were washed and incubated at 37 °C. At 0, 3, 6, 12, 24, and 48 h post-infection (hpi), cells and supernatants were harvested and assayed for SARS-CoV-2 total and neg-RNA content. These experiments were performed in a BSL3 facility.

### 2.4. PBMC Infection 

PBMC were obtained from healthy donors by Ficoll/Hypaque (Pharmacia, Sweden) density centrifugation. Cultures were performed in RPMI1640 medium (GIBCO, Waltham, MA, USA) containing 10% heat-inactivated FCS. PBMC from 8 donors were used in the infection experiments. For these experiments, PBMC were exposed to 2019-nCoV/Italy-INMI1 isolate for 1 h at 37 °C at MOI of 1; at the end of the adsorption period, PBMC were washed, reseeded at 2 × 10^6^ cells/mL in RPMI 10% FCS, and incubated at 37 °C. At 0, 12, 24, 48 and 72 hpi, supernatant and cells were collected and stored at −80 °C for subsequent analysis. These experiments were performed in BSL3 facility.

### 2.5. Viral RNA Amplification

Total-RNA was extracted from VeroE6 cells and PBMC using Trizol (Life Technologies, Grand Island, NY, USA) and from VeroE6 and PBMC supernatants using QIAamp^®^ Viral RNA Mini Kit (Qiagen, Hilden, Germany), according to the manufacturer’s instructions. SARS-CoV-2 total-RNA was amplified via real-time quantitative RT-PCR (qRT-PCR) in a Rotor-GeneQ Real-Time cycler (Qiagen, Hilden, Germany). The SuperScript^®^ III One-Step RT-PCR System kit (Invitrogen, Karlsruhe, Germany) was used with a 25 µL reaction mixture under the following conditions: 0.5 µL of kit enzyme mixture, 12.5 µL of 2X Reaction Mix, 0.8 µL of MgSO4, 0.5 µL of 25 µM primer mix, 0.5 μL of 20 µM of probe, 4.7 μL of nuclease-free water (Mol Biograde, Hamburg, Germany), and 5 µL of the extracted sample. The following thermal profile was used: a cycle of reverse transcription of 30 min at 50 °C 2 min at 95 °C for reverse transcriptase inactivation and DNA polymerase activation followed by 45 amplification cycles of 15 s at 95 °C and 1 min at 60 °C. The primers and probe sequences are described elsewhere [26]. 

To measure neg-RNA, the reverse transcription step was minus strand-specific, based on the use of the forward primer only, as described for the detection of other viral RNAs [27,28]. cDNAs were treated with 1 µL of RNase H (20 U/µL) for 20′ at 37 °C, to remove viral RNAs, and cDNA was amplified with the SuperScript^®^ III One-Step RT-PCR System kit, with a modified thermal profile, that omitted the reverse transcription step. 

### 2.6. IFN and Cytokine Detection

IFN-α released in PBMC supernatants and present in plasma samples from COVID-19 patients was measured by enzyme-linked immunosorbent assay (ELISA) VeriKine-HS Human IFN-α purchased from PBL Assay Science (Piscataway, NJ, USA). Results were expressed as pg/mL. The detection range was 1.95–125 pg/mL for IFN-α. As a positive control for IFN induction, Newcastle Disease Virus (NDV) was used in all the experiments, at 10 hemagglutination units (HAU)/10^6^ cells. In all cases, a strong response was observed in NDV-exposed PBMC, indicating good inducibility of IFN-α in our experimental conditions. As a negative control for cytokine detection, PBMCs treated with only medium were used.

Inflammatory cytokine IL8 in culture’s supernatants was quantified using automated multiplex immunoassay on Ella instrument (San Jose, CA, USA). The detection limit 0.19 pg/mL for IL8.

### 2.7. Statistical Analysis

Inferential analysis of association was performed using Mann–Whitney or Kruskal–Wallis tests for continuous parameters. Analyses were performed using GraphPad Prism version 8.00 (GraphPad Software, La Jolla, CA, USA) and SPSS version 23 for Windows statistical software; a *p*-value < 0.05 was considered statistically significant.

### 2.8. Ethical Statement

The study was conducted in accordance with the Declaration of Helsinki and with protocol code n°. 70, and was approved on 17 December 2018 by the institutional review board of the National Institute for Infectious Diseases, L. Spallanzani, IRCCS, according to which the study protocol described here did not require the signing of informed consent by the patients because no further samples were taken other than those obtained for diagnostic purposes. The data of biological samples collected for diagnostic purposes were used only after their complete anonymization. Furthermore, the analysis of genetic data was not provided.

## 3. Results

### 3.1. In Vitro Experiments for Viral Replication

**Figure 1:**
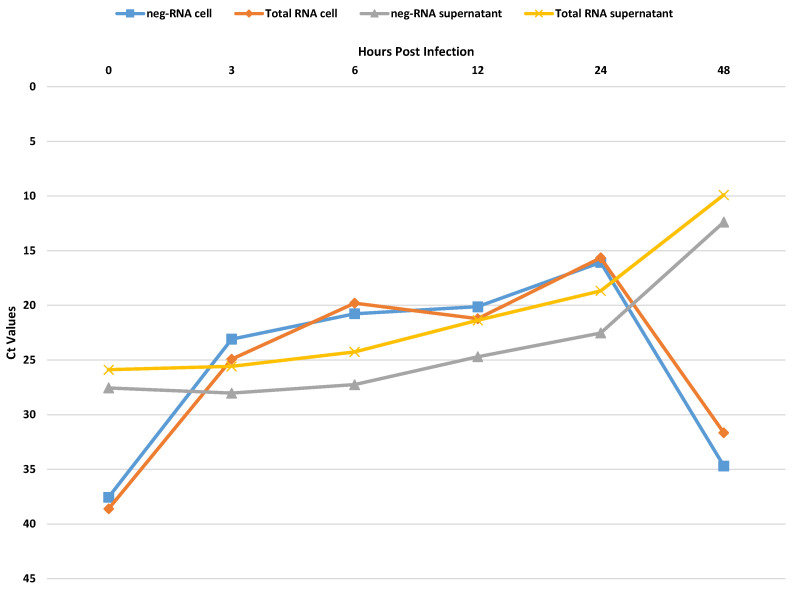
Distribution of SARS-CoV-2 RNA types in infected cells: Vero E6 cells were exposed to 2019-nCoV/Italy-INMI1 isolate for 1 h at 37 °C at MOI of 0.1; at 0, 3, 6, 12, 24 and 48 hpi, cell pellets and supernatants were collected and the levels of total and negative strand RNA, either cell-associated or shed in the culture supernatant, were measured.

We set up and evaluated a specific method to measure SARS-CoV-2 neg-RNA strand, which is a surrogate marker of ongoing viral replication, performing preliminarily experiments related to in vitro infection on Vero E6 cells. As shown in Figure 1, cell-associated SARS-CoV-2 total- and neg-RNA started to increase after 3 hpi and peaked at 24 hpi, whereas in the supernatants, SARS-CoV-2 total- and neg-RNA were detectable at 0 and 6 hpi, slightly increased at 12 hpi, and peaked at 48 hpi. The neg-RNA was mostly cell-associated until 24 hpi, and the cells/supernatants ratio reversed after 48 hpi when the cytopathic effect was maximal, confirming the good performance of the set-up method (Figure 1). 

When we extended the analysis to PBMC from healthy donors exposed to SARS-CoV-2, no viral replication was observed. In fact, cell-associated RNA levels, either total or neg-RNA, remained unchanged compared to the inoculum throughout the whole study period, while decreasing amounts of viral RNA (both total and negative) were measured in supernatants after 12 hpi, suggesting the presence of residual inoculated virus rather than replicative SARS-CoV-2.

### 3.2. IFN Induction and IL-8 Levels in PBMC Infected with SARS-CoV-2

**Figure 2:**
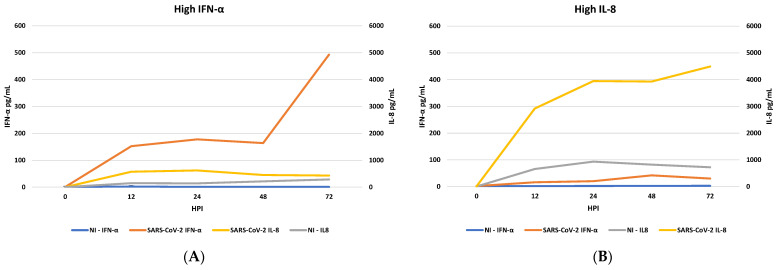
Induction of Interferon alpha (IFN-α) immunoreactive cytokines and IL-8 chemokines by live SARS-CoV-2. Peripheral Blood Monocyte Cells (PBMC) were infected with SARS-CoV-2 at a Molteplicity of infection (MOI) 1. At different hours post infection (hpi) (0, 12, 24, 48, and 72) released cytokines were detected by Enzime Linked Immuno Sorbent Assay (ELISA) in both SARS-CoV-2 infected PBMC and non-infected PBMC and expressed as pg/mL. Results are grouped in IFN-α high levels ((**A**), results are representative of four independent experiments) and IL-8 high levels ((**B**), results are representative of four independent experiments).

To test the ability of SARS-CoV-2 to induce IFN response and produce IL-8 in PBMC, cells from eight different healthy donors were exposed to SARS-CoV-2 INMI isolate at MOI of 1. Supernatants of PBMC-infected cultures were collected at different time points (0, 12, 24, 48, and 72 hpi) and the production of either IFN-α or IL-8 was assessed. Interestingly, neither IFN-α nor IL-8 induction was the same in all the challenged PBMC. In fact, in those PBMC with medium or high IL-8 levels (>1000 pg/mL), IFN-α levels were low (<100 pg/mL). Figure 2 shows the trend of IFN-α and IL-8 at different time points from infection in 8 PBMC challenged with SARS-CoV-2. In more detail, in four of them, IFN-α increased over time, with an increasing trend at 72 hpi, while IL-8 levels remained constantly low at all time points (Figure 2A). On the contrary, in the remaining four PBMC, IL-8 levels rapidly increased after 12 hpi, whereas IFN-α levels remained constantly low at all time points (Figure 2B). 

### 3.3. IFN-α and IL-8 Levels in Plasma Samples from COVID-19 Patients

**Figure 3:**
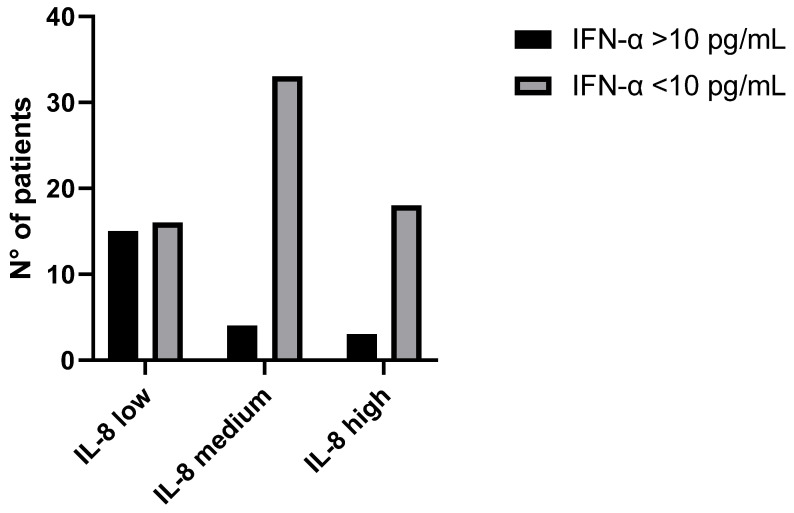
Evaluation of IFN-α and IL-8 levels in plasma samples from COVID-19 patients, divided into three groups based on the IL-8 levels: 31 patients with IL-8 low (<100 pg/mL), among which 16 have IFN-α levels < 10 pg/mL and 15 have IFN-α levels > 10 pg/mL; 37 patients with IL-8 medium (101 < pg/mL < 1000), among which 33 have IFN-α levels < 10 pg/mL and 4 have IFN-α levels > 10 pg/mL; 21 patients with IL-8 high (>1001 pg/mL), among which 18 have IFN-α levels < 10 pg/mL and 3 have IFN-α levels > 10 pg/mL The interrelation between IFN-α low levels (<10 pg/mL) and high levels (>10 pg/mL) and the three groups is shown in the histogram, with a statistically significant correlation (*p* < 0.001) between IFN-α low levels and IL-8 medium and high groups and IFN-α high levels and IL-8 low group.

In order to confirm the possible antagonistic effect between IFN-α and IL-8 in SARS-CoV-2 infected patients, a total of 89 plasma samples from COVID-19 patients were tested for the IFN-α and IL-8 levels. Patients were divided based on their IL-8 levels into three groups: IL-8 low (<100 pg/mL), IL-8 medium (101 < pg/mL < 1000) and IL-8 high (>1001 pg/mL). Figure 3 shows the interrelation between IFN-α and IL-8 levels in the three groups: high levels of IFN-α (>10 pg/mL) were evidenced only in the IL-8 low group; conversely, IL-8 medium and high groups correlated with lower levels of IFN-α (<10 pg/mL). The correlation between low levels of IFN-α and high levels of IL-8 was statistically significant (*p* < 0.001) in both the IL-8 medium and IL-8 high group. 

**Figure 4:**
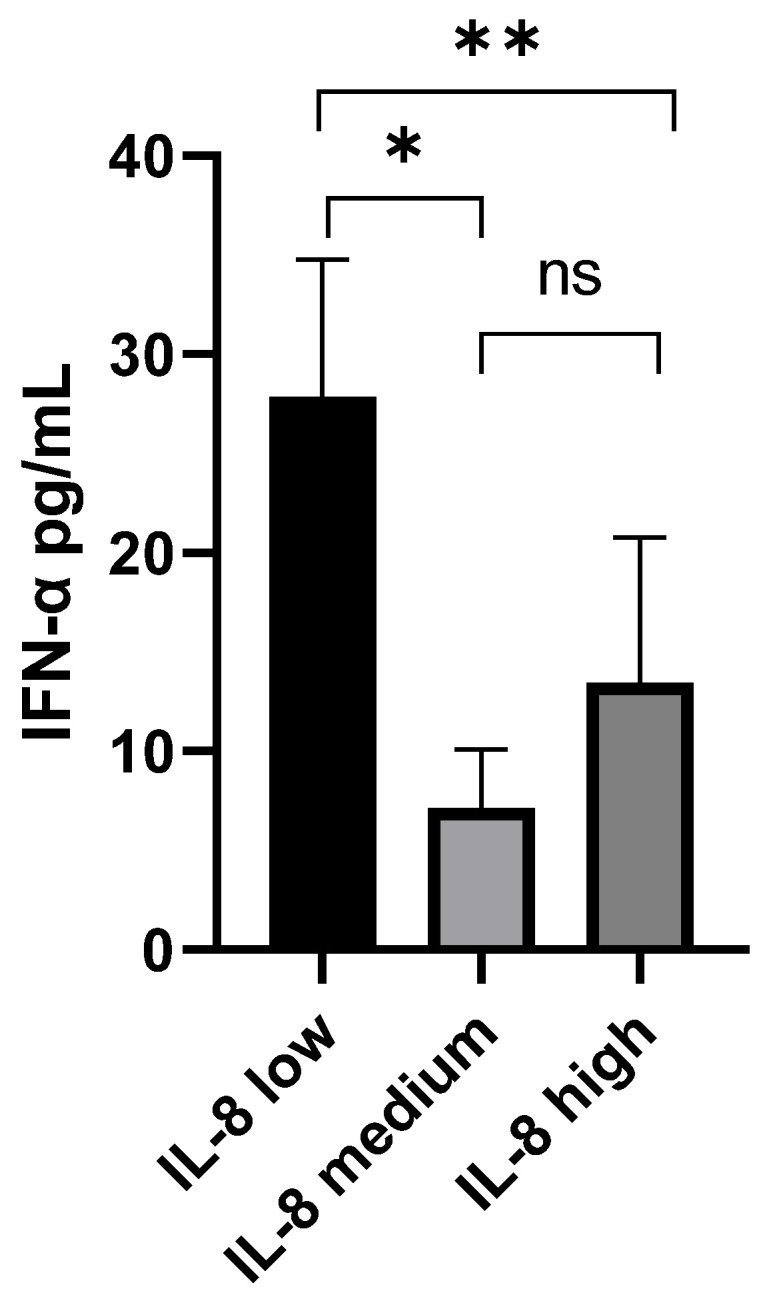
Kruskal–Wallis test between IFN-α and IL-8 groups: median values and standard errors are shown. A statistically significant difference was found between IL-8 low and IL-8 groups (*: *p* < 0.05) and between the IL-8 low and IL-8 high groups (**: *p* < 0.001). No statistically significant (ns) difference was found between the IL-8 medium and IL-8 high groups.

Moreover, Figure 4 shows the differences between the three groups as compared to IFN-α levels evaluated through the Kruskal–Wallis test: a statistically significant difference was present between the IL-8 low vs. the IL-8 medium groups (*p* < 0.05) and between the IL-8 low and the IL-8 high groups (*p* < 0.01); no significant difference was present between the IL-8 medium and IL-8 high groups.

## 4. Discussion

Since the emergence of SARS-CoV-2 infection, the global pandemic of COVID-19 has caused over 6.8 million deaths among over 760 million confirmed cases worldwide [1]. Disease progression greatly varies in COVID-19: patients may be asymptomatic, have mild symptoms or develop severe and fatal disease. However, it is difficult to predict the disease progression and outcome of SARS-CoV-2 infection and there have been many efforts to better understand and identify factors that may lead to a fatal outcome and help clinicians develop countermeasures [29,30]. A paper by Li et al. [21] compared the cytokine levels in the serum samples of COVID-19 patients at longitudinal time points during severe illness or at recovery and identified differential cytokine profiles potentially associated with COVID-19 disease status [21]. Besides IL-6, which has already been reported as a potential biomarker for COVID-19 patients [29,30], they identified that both IL-6 and IL-8 serum levels were elevated in COVID-19 patients with severe diseases. Interestingly, IL-8 levels correlated better than IL-6 with the overall clinical disease scores at the different time points in the same COVID-19 patients [21]. As in other infections, an antagonistic effect between type I IFN and IL-8 has been highlighted [22,23,24], and we evaluated if it may also play a role in SARS-CoV-2 infection. 

Notably, our study showed a probable antagonistic effect between IL-8 and IFN-α in PBMC from healthy donors challenged with SARS-CoV-2 and that productive infection of PBMC is not mandatory for cytokines induction. Moreover, we observed inversely proportional IFN-α levels to IL-8 concentrations detected in plasma samples from COVID-19 patients. Specifically, a statistically significant difference was evidenced in IFN-α expression between the IL-8 low and the IL-8 medium and IL-8 high groups. Moreover, a statistically significant correlation was present between patients with low levels of IFN-α and high levels of IL-8 and between patients with high levels of IFN-α and low levels of IL-8. In COVID-19 patients, type I IFN signaling is required for ISG (interferon-stimulated gene) induction and the recruitment of pro-inflammatory cells in the lung, indicating its necessary role in host defense against viral infection. Furthermore, a decrease in type I IFN-related immunity may cause severe progression in COVID-19 patients [31], indicating that the effective activation of innate immunity, mainly through type I IFN responses and their downstream cascades, is essential to suppress and eliminate viral replication during SARS-CoV-2 infection [32]. As stated above, IL-8 has been associated with increased disease severity and our findings regarding the cross-talk and/or antagonistic effect between IFN-α and IL-8 may suggest that the impairment of the innate immune response in COVID-19 patients may be linked to a dysregulated cytokine response, namely through IL-8 production. A limitation of the study is that no data concerning the severity, the eventual hospitalization in intensive care unit, or the onset of complications in COVID-19 patients are available; therefore, it was not possible to make any association between laboratory findings and clinical presentation/outcome.

Further studies are needed to better understand the antagonistic effect and the possible downstream consequences on disease severity, as well as to identify possible new therapeutic targets.

## Data Availability

The data used and/or analyzed during the study are available from the corresponding author on reasonable request.

## References

[1] World Health Organization. https://covid19.who.int/.

[2] Huang C., Wang Y., Li X., Ren L., Zhao J., Hu Y., Zhang L., Fan G., Xu J., Gu X. (2020). Clinical features of patients infected with 2019 novel coronavirus in Wuhan, China. Lancet.

[3] Karki R., Kanneganti T.-D. (2021). The ‘cytokine storm’: Molecular mechanisms and therapeutic prospects. Trends Immunol..

[4] Zheng Z., Peng F., Xu B., Zhao J., Liu H., Peng J., Li Q., Jiang C., Zhou Y., Liu S. (2020). Risk factors of critical & mortal COVID-19 cases: A systematic literature review and meta-analysis. J. Infect..

[5] Karki R., Kanneganti T.-D. (2022). Innate immunity, cytokine storm, and inflammatory cell death in COVID-19. J. Transl. Med..

[6] Capobianchi M.R., Uleri E., Caglioti C., Dolei A. (2015). Type I IFN family members: Similarity, differences and interaction. Cytokine Growth Factor Rev..

[7] Gao Y., Li T., Han M., Li X., Wu D., Xu Y., Zhu Y., Liu Y., Wang X., Wang L. (2020). Diagnostic utility of clinical laboratory data determinations for patients with the severe COVID-19. J. Med. Virol..

[8] Mehta P., McAuley D.F., Brown M., Sanchez E., Tattersall R.S., Manson J.J., HLH Across Speciality Collaboration, UK (2020). COVID-19: Consider cytokine storm syndromes and immunosuppression. Lancet.

[9] Manjili R.H., Zarei M., Habibi M., Manjili M.H. (2020). COVID-19 as an Acute Inflammatory Disease. J. Immunol..

[10] Kindler E., Thiel V. (2016). SARS-CoV and IFN: Too Little, Too Late. Cell Host Microbe.

[11] Castilletti C., Bordi L., Lalle E., Rozera G., Poccia F., Agrati C., Abbate I., Capobianchi M.R. (2005). Coordinate induction of IFN-α and -γ by SARS-CoV also in the absence of virus replication. Virology.

[12] Blanco-Melo D., Nilsson-Payant B.E., Liu W.-C., Uhl S., Hoagland D., Møller R., Jordan T.X., Oishi K., Panis M., Sachs D. (2020). Imbalanced Host Response to SARS-CoV-2 Drives Development of COVID-19. Cell.

[13] Lucas C., Wong P., Klein J., Castro T.B.R., Silva J., Sundaram M., Ellingson M.K., Mao T., Oh J.E., Israelow B. (2020). Longitudinal analyses reveal immunological misfiring in severe COVID-19. Nature.

[14] Galani I.-E., Rovina N., Lampropoulou V., Triantafyllia V., Manioudaki M., Pavlos E., Koukaki E., Fragkou P.C., Panou V., Rapti V. (2021). Untuned antiviral immunity in COVID-19 revealed by temporal type I/III interferon patterns and flu comparison. Nat. Immunol..

[15] Pasrija R., Naime M. (2021). The deregulated immune reaction and cytokines release storm (CRS) in COVID-19 disease. Int. Immunopharmacol..

[16] Zhang C., Wu Z., Li J.-W., Zhao H., Wang G.Q. (2020). Cytokine Release Syndrome in Severe COVID-19: Interleukin-6 Receptor Antagonist Tocilizumab may be the Key to Reduce Mortality. Int. J. Antimicrob. Agents.

[17] Kuppalli K., Rasmussen A.L. (2020). A glimpse into the eye of the COVID-19 cytokine storm. EBioMedicine.

[18] Moore B.J.B., June C.H. (2020). Cytokine release syndrome in severe COVID-19. Science.

[19] Zhong J., Tang J., Ye C., Dong L. (2020). The immunology of COVID-19: Is immune modulation an option for treatment?. Lancet Rheumatol..

[20] Soy M., Keser G., Atagündüz P., Tabak F., Atagündüz I., Kayhan S. (2020). Cytokine storm in COVID-19: Pathogenesis and overview of anti-inflammatory agents used in treatment. Clin. Rheumatol..

[21] Li L., Li J., Gao M., Fan H., Wang Y., Xu X., Chen C., Liu J., Kim J., Aliyari R. (2021). Interleukin-8 as a Biomarker for Disease Prognosis of Coronavirus Disease-2019 Patients. Front. Immunol..

[22] Aman M.J., Rudolf G., Goldschmitt J., Aulitzky W.E., Lam C., Huber C., Peschel C. (1993). Type-I interferons are potent inhibitors of interleukin-8 production in hematopoietic and bone marrow stromal cells. Blood.

[23] Choi A.M., Jacoby D.B. (1992). Influenza virus A infection induces interleukin-8 gene expression in human airway epitheial cells. FEBS Lett..

[24] Khabar K.S., Al-Zoghaibi F., Al-Ahdal M.N., Murayama T., Dhalla M., Mukaida N., Taha M., Al-Sedairy S.T., Siddiqui Y., Kessie G. (1997). The α Chemokine, Interleukin 8, Inhibits the Antiviral Action of Interferon α. J. Exp. Med..

[25] Reed L.J., Muench H.A. (1938). A simple method of estimating fifty percent endpoints. Am. J. Hyg..

[26] Corman V.M., Landt O., Kaiser M., Molenkamp R., Meijer A., Chu D.K.W., Bleicker T., Brünink S., Schneider J., Schmidt M.L. (2020). Detection of 2019 novel coronavirus (2019-nCoV) by real-time RT-PCR. Eurosurveillance.

[27] Biava M., Caglioti C., Bordi L., Castilletti C., Colavita F., Quartu S., Nicastri E., Lauria F.N., Petrosillo N., Lanini S. (2017). Detection of Viral RNA in Tissues following Plasma Clearance from an Ebola Virus Infected Patient. PLOS Pathog..

[28] Biava M., Caglioti C., Castilletti C., Bordi L., Carletti F., Colavita F., Quartu S., Nicastri E., Iannetta M., Vairo F. (2018). Persistence of ZIKV-RNA in the cellular fraction of semen is accompanied by a surrogate-marker of viral replication. Diagnostic implications for sexual transmission. New Microbiol..

[29] Maeda T., Obata R., Do D.R., Kuno T. (2021). The association of interleukin-6 value, interleukin inhibitors, and outcomes of patients with COVID-19 in New York City. J. Med. Virol..

[30] Laguna-Goya R., Utrero-Rico A., Talayero P., Lasa-Lazaro M., Ramirez-Fernandez A., Naranjo L., Segura-Tudela A., Cabrera-Marante O., de Frias E.R., Garcia-Garcia R. (2020). IL-6–based mortality risk model for hospitalized patients with COVID-19. J. Allergy Clin. Immunol..

[31] Zhang Q., Bastard P., Liu Z., Le Pen J., Moncada-Velez M., Chen J., Ogishi M., Sabli I.K.D., Hodeib S., Korol C. (2020). Inborn errors of type I IFN immunity in patients with life-threatening COVID-19. Science.

[32] Yang L., Wang J., Hui P., Yarovinsky T.O., Badeti S., Pham K., Liu C. (2021). Potential role of IFN-α in COVID-19 patients and its underlying treatment options. Appl. Microbiol. Biotechnol..

